# The efficacy of essential oil mixtures on vase life and the quality of Gerbera cut flowers

**DOI:** 10.1038/s41598-025-22152-6

**Published:** 2025-10-27

**Authors:** Safia Hamdy El-Hanafy, Mayada Muhammad Najuib Ahmad

**Affiliations:** 1 Department of Ornamental Horticulture, Faculty of Aquaculture, Cairo University, Giza, Egypt; 2 Department of Ornamental Plant and Woody Trees, Agricultural and Biological Research Institute, National Research Centre (NRC), P.O. Box 12622, Dokki, Giza, Egypt

**Keywords:** *Gerbera jamesonii*, Essential oils, Mixtures, Cut flowers, Postharvest, Preservatives, Biochemistry, Biotechnology, Plant sciences

## Abstract

Gerbera is one of the four most important cut flowers worldwide, ranking fourth among cut flowers beyond roses, chrysanthemums, and tulips. Utilizing essential oils (EOs) as preservative material to control bacterial and fungal contamination, as well as to reduce postharvest quality loss in several cut flowers is a necessity in recent days. It was thought of investigating mixtures of essential oils to maximize their benefits in preserving cut flowers. Four different mixtures of essential oils were examined to preserve *Gerbera jamesonii* L. cv. Froza cut inflorescence. They were Cumin oil (150 µlL^− 1^) and Peppermint oil (150 µlL^− 1^) [T2], Cumin oil (150 µlL^− 1^) and Nigella oil (150 µlL^− 1^) [T3], Clove oil (150 µlL^− 1^) and Anise oil (150 µlL^− 1^) [T4], and Lavender oil (150 µlL^− 1^) and Thyme oil (150 µlL^− 1^) [T5], while the control cut inflorescences were held in distilled water and 0.5 µlL^− 1^ of tween-20 [T1]. The utilized mixtures effectively prolonged the vase life of Gerbera cut inflorescences. They also maintained the fresh and dry weights of the cut inflorescences, enhanced the water relations, raised the inflorescence and scape diameters, and preserved the total contents of anthocyanins and carbohydrates. They dramatically decreased the growth of microorganisms in the vase solution. The combination of cumin oil and nigella oil was the most effective mixture in most of the studied characteristics. This mixture could extend the vase life of Gerbera cut inflorescences by approximately 7 days, rather than the control. Applying various mixtures of essential oils is a novel field that requires further studies and analysis.

## Introduction

 Gerbera (*Gerbera jamesonii* L.) is a perennial herb that is related to the Asteraceae family. It is commercially produced worldwide. It has many names, such as Transvaal Daisy, Barberton Daisy, or Veldt Daisy. It has superfine inflorescence, which is colored radiantly^1^. It is known by its large flower heads, which are distinguished by their ray-like petals, which are aligned around a central disk of teeny green or black flowers. A long, leafless, and vertical scape upholds the inflorescence. Gerbera has radical lanceolate leaves, which are strongly lobed, leathery at times, narrower at the base, and broader at the top^2^. Gerbera has many uses: floral arrangements, decorations, bouquet making, and flower vases. Recently, numerous cultivars of gerbera are available in distinct colors and characteristics in markets. Some parameters determine the market demand and price of the product, such as flower color, quality, and vase life of gerbera^1^. Gerbera ranks fourth among the cut flowers, beyond roses, chrysanthemums, and tulips^2^. Gerbera cut inflorescences have a relatively short vase life. This is attributed to the low lignin content of the scape, which makes the flower quite perishable. Moreover, they are susceptible to microbial contamination of the preservative solution, which causes stem end blockage. This leads to a disequilibrium between the water absorbed and the water lost, ultimately resulting in wilting of the inflorescences and shortening of the vase life. Another main reason for the gerbera’s short vase life is the stem hollowness, which is a result of high humidity and high temperature^2^.

Utilizing natural and safe substances such as essential oils (EOs) to control bacterial and fungal contamination, as well as to reduce postharvest quality loss in several cut flowers, is a necessity in recent days. Essential oils extracted from aromatic plants consist of organic substances, such as terpenoids and their oxygenated derivatives, in addition to the phenolic compounds, which are mainly responsible for their antibacterial properties^3^. Moreover, EOs efficiently increased the absorbed water, reduced the lost water, enhanced quality parameters, and improved the content of many biochemical constituents of numerous cut flowers in various research over the last seventeen years. Cumin oil had positive effects on relative fresh weight, ratio of fresh weight to dry weight of petals, flower diameter, stem diameter, water content, soluble sugar content, the absorption rate of the solution, and anthocyanin content of rose cut flowers^4^. Peppermint oil prolonged the vase life, improved flower quality by helping preserve petal membrane stability and petal anthocyanin content, and reduced microbial cell count in the xylem vessels of *Dendrobium* orchids^5^. Nigella oil increased the vase life, final water uptake, and shoot fresh weight/shoot dry weight ratio, while it decreased the loss of flower fresh weight and the number of bacterial colonies in the vase solution^6^. Clove oil efficiently prolonged the vase life, as well as increased solution uptake, fresh and dry weights, chlorophylls, and total sugars. On the contrary, it suppressed the growth of microorganisms in the vase solution of chrysanthemum cut flowers^7^. Thyme oil doubled the vase life of carnation cut flowers, compared to the control. Moreover, thyme and anise oils increased the water uptake, yet they restricted the severe decline of chlorophyll and total carbohydrate content of carnation cut flowers. Furthermore, anise oil inhibited the microbial growth and prevented the base of carnation stems from blockage for nine days in the vase solution^8^.

This study was established to emphasize the positive impacts of EOs on preserving cut flowers by using different mixtures of them. The applied mixtures were: (a) cumin oil and peppermint oil, (b) cumin oil and nigella oil, (c) clove oil and anise oil, and (d) lavender oil and thyme oil. These mixtures were used for elongating the vase life of *Gerbera jamesonii* L. cv. Froza cut inflorescences. They also applied because of their potential abilities to enhance water relations, improve the quality of the inflorescences, preserve their anthocyanin pigment content, increase their carbohydrate content, and diminish the microbial growth in the vase solution.

## Materials and methods

### Location and duration

The experiment was consummated during the period from 26^th^ of November to 22^nd^ of December, 2024, at laboratory of Ornamental Plants and Woody Trees Department, National Research Centre, Dokki, Giza, Egypt.

### Plant material

The commercial nursery (Floramix Farm) in Giza, Egypt was the source of *Gerbera jamesonii* L. cv. Froza cut inflorescences. They were harvested when they were mature and had full opening. They were pre-cooled immediately to mitigate the effect of high field temperature (the stalks were placed into buckets with holding solution soon after harvesting, then these buckets were transferred to a pre-cooling chamber with a temperature of approximately 3–4 °C). After that, they were wrapped in Kraft paper in groups of 10 inflorescences for each. The inflorescences were transported directly from the farm to the laboratory. The ends of gerbera inflorescence were re-cut under tap water to a standard length of 50 cm in order to avert air embolism.

### Essential oils

The applied essential oils in this experiment were obtained from “Squeezing and Extracting Natural Oils Unit”, National Research Centre, Dokki, Giza. They were extracted by steam distillation method.

### Procedures and treatments

Every inflorescence was placed in a graduated cylinder having 500 ml of holding solution. The inflorescences were split as follows:

- Control (distilled water and 0.5 µlL^− 1^ of tween-20) [T1].
- Cumin oil (150 µlL^− 1^) and Peppermint oil (150 µlL^− 1^) [T2].
- Cumin oil (150 µlL^− 1^) and Nigella oil (150 µlL^− 1^) [T3].
- Clove oil (150 µlL^− 1^) and Anise oil (150 µlL^− 1^) [T4].
- Lavender oil (150 µlL^− 1^) and Thyme oil (150 µlL^− 1^) [T5].

These essential oils were dissolved in 0.5 µlL^− 1^ of tween-20 then added to the distilled water^9^.

The layout of the experiment was completely randomized design. Each treatment had four replicates and one cut inflorescence was used for each replicate. The control inflorescence was placed in distilled water with 0.5 µlL^− 1^ of tween-20.

### Assessment of vase life

#### Vase life of the cut inflorescences (days)

This was recorded from the day of harvesting to the day of senescence.

#### Relative fresh weight (R.F.W. %)

The cut inflorescences were weighed day after day through the vase duration. The relative changes (in relation to the fresh weight on the day of harvesting) were figured out by the formula: R.F.W. (%) = (W_t_/W_t=0_)×100; whereas, W_t_ is the fresh weight of the inflorescence in grams at a specific day (t = 3, 5, 7, etc.) while W_t=0_ is the fresh weight of the inflorescence on the harvesting day in grams^10^.

#### Dry matter percentage (D.M. %)

The fresh weight of each inflorescence was weighed by digital balance at the end of the vase life. After that, the inflorescences were dried in oven at 70 °C, for 24 h. the dry matter percentage was measured by the following formula: D.M. (%) = (dry weight/fresh weight)*100^11^.

### Water relations

#### Total and daily water uptake (ml scape^− 1^ day^− 1^)

The readings of the graduated cylinders were recorded day after day through the vase period. The total water uptake was the aggregate of the water uptake of the inflorescence during the vase life. The daily water uptake was calculated by dividing the total water uptake by the days that the inflorescence had lived.

#### Total and daily water loss (g scape^− 1^ day^− 1^)

The weight of cylinders (WC) and the weight of inflorescences (WF) were registered every two days. Water loss was calculated by the formula: Water loss = (WC_t_-WC_t+2_)-(WF_t_-WF_t−2_); where, WC_t_ is the cylinder weight at day 0, 3, 5, etc., WC_t+2_ is the cylinder weight after two days, WF_t_ is the inflorescence weight at day 3, 5, 7, etc., and WF_t−2_ is the weight of inflorescence after two days. The total and the daily water loss were calculated as was shown above in the total and the daily water uptake.

### Assessment of inflorescence quality

#### The diameter decreasing index of scape (SDDI) and inflorescence (IDDI)

The scape and inflorescence diameters were measured every other day with a vernier caliper; SDDI as well IDDI were calculated as following expression: D_3_/D_1_ + D_5_/D_3_+…+D_f−2_/D_f_, whereas; D is scape or inflorescence diameter, and D_f_ is last day scape or inflorescence diameter. The number of this formula was divided to the number of ratios and the final number represents the inflorescence diameter decreasing index^12^.

### Biochemical analysis

#### Total anthocyanin content (g/100 g of fresh ray flowers)

Anthocyanin content in fresh ray flowers was determined on the 8^th^ day by colorimetric method according to Husia et al. 1965^13^. Fresh samples of ray flowers (0.5 g) were soaked in ethyl (95% v/v) acidic with concentration hydrochloric acid (1 mm) for 16 h away from the light. The extract was filtered using a glass funnel (G4). The residue was washed several times with the solvent until the filtrate become colorless. The combined extract was completed to a known volume (10 ml). A small quantity of this extract was taken for the colorimetric determination of pigments, using a spectrophotometer at wave length of 535 nm. Ethyl (95% v/v) acidic with concentration hydrochloric acid (1 mm) was used as standard blank.

#### Total carbohydrate content (percentage Of D.W. Of the inflorescence)

The total carbohydrate content was determined using the phenol-sulphuric acid method, according to Dubois et al. (1956)^14^.

### Biological studies

#### Total plate count of microorganisms in preservation solution (Colony forming unit CFU/ml)

Samples of 3 ml of the vase solutions were taken on the 8^th^ day of the vase life of the inflorescences to undergo microbial analysis. The microbiology analysis was performed in Cairo University Research Park (CURP), Faculty of Agriculture, Cairo University. The standard method and suitable culture media were used for the microbiological analysis. The method used was according to ISO 4833:2033 (E) Microbiology of food and animal feeding stuffs – Horizontal method for the enumeration of microorganisms – colony count technique at 30 °C.

### Statistical analysis

Means were compared for significance by Duncanʼs New Multiple Range test at 0.05% level of probability using COSTATV-63^15^. Standard error was calculated by Microsoft Excel Worksheet 2010.

## Results

### Assessment of vase life

#### Vase life of the cut inflorescences (days)

The applied essential oil mixtures caused significant elongation of the gerbera span life compared to the control (10.75 days). The mixture of cumin oil and nigella oil was the most effective mixture which recorded 17.50 days (Table 1).

**Table 1:** Vase life, R.F.W. %, D.M. %, and water relations affected by different EOs mixtures added to the vase solution of *Gerbera Jamesonii* L. c.v. Froza.

Treatments	Vase Life (days)	*R*.F.W. %	D.M. %	Total Water Uptake (ml inflorescence^− 1^)	Daily Water Uptake ml inflorescence ^− 1^ day^− 1^)	Total Water Loss (g inflorescence^− 1^)	Daily Water Loss (g inflorescence^− 1^ day^− 1^)
T1	10.75^d^ ± 0.75	102.38^d^ ± 0.65	6.00^d^ ± 0.34	20.00^d^ ± 2.04	1.86^c^ ± 0.12	20.27^d^ ± 2.63	1.87^c^ ± 0.14
T2	15.50^b^ ± 0.50	108.47^d^ ± 0.99	10.00^c^ ± 0.52	85.00^a^ ± 8.42	5.45^a^ ± 0.35	86.25^a^ ± 8.75	5.53^a^ ± 0.37
T3	17.50^a^± 0.50	156.85^a^ ± 6.67	16.35^a^ ± 0.18	71.25^ab^ ± 4.73	4.06^b^ ± 0.15	67.56^b^ ± 5.23	3.85^b^ ± 0.19
T4	13.00^**c**^ ± 0.00	140.36^b^ ± 2.70	9.32^c^ ± 0.49	50.00^c^ ± 2.04	3.85^b^ ± 0.16	47.39^**c**^ ± 2.03	3.65^b^ ± 0.16
T5	13.50^c^± 0.50	128.60^c^ ± 3.50	14.11^b^ ± 0.49	57.50^bc^ ± 2.50	4.26^b^ ± 0.03	55.36^bc^ ± 2.76	4.09^b^ ± 0.05

The means inside a column with the same letters are not significantly different according to Duncan’s Multiple Range Test (at the 0.05% level). [T1]: Control (distilled water and 0.5 µlL^− 1^ of tween-20); [T2]: Cumin oil (150 µlL^− 1^) and Peppermint oil (150 µlL^− 1^); [T3]: Cumin oil (150 µlL^− 1^) and Nigella oil (150 µlL^− 1^); [T4]: Clove oil (150 µlL^− 1^) and Anise oil (150 µlL^− 1^); [T5]: Lavender oil (150 µlL^− 1^) and Thyme oil (150 µlL^− 1^). R.F.W.%; Relative Fresh Weight%; D.M.%: Dry Matter %.

#### Relative fresh weight (R.F.W. %)

Data in Table 1 represent R.F.W. % on the 10^th^ day. The mixture of cumin oil and nigella oil had the highest impact on the R.F.W. %, as it significantly increased the fresh weight of the inflorescence by 156.85% relative to its weight in zero time, while the control cut inflorescences recorded 102.38%.

#### Dry matter (D.M. %)

Essential oil mixtures statistically raised the percentage of dry matter more than the control cut inflorescences, with a superiority to the mixture of cumin oil and nigella oil (Table 1).

### Water relations

The utilizing mixtures recorded significant increment in both water uptake and water loss (total and daily) in comparison with the control. The mixture of cumin oil and peppermint oil recorded the highest values in total and daily water uptake, as well as total and daily water loss, whereas the control inflorescences had the lowest values (Table 1).

### Assessment of inflorescence quality

#### The diameter decreasing index of scape (SDDI) and inflorescence (IDDI)

**Fig. 1:**
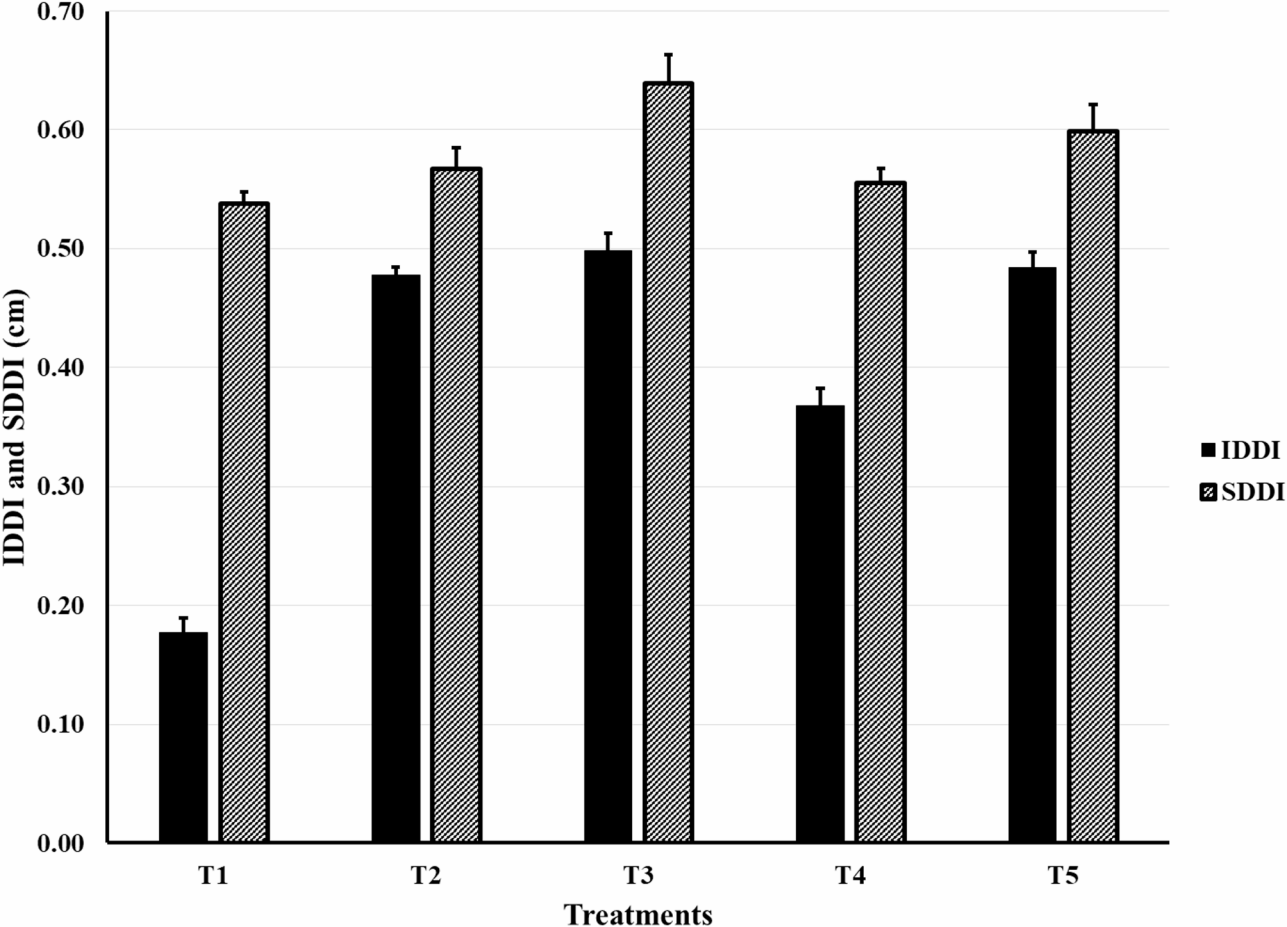
IDDI and SDDI affected by different EOs mixtures added to the solution of *Gerbera jamesonii* L. c.v. Froza. [T1]: Control (distilled water and 0.5 µlL^− 1^ of tween-20); [T2]: Cumin oil (150 µlL^− 1^) and Peppermint oil (150 µlL^− 1^); [T3]: Cumin oil (150 µlL^− 1^) and Nigella oil (150 µlL^− 1^); [T4]: Clove oil (150 µlL^− 1^) and Anise oil (150 µlL^− 1^); [T5]: Lavender oil (150 µlL^− 1^) and Thyme oil (150 µlL^− 1^). SDDI: Diameter Decreasing Index of Scape; IDDI: Diameter Decreasing Index of Inflorescence.

All applied mixtures boosted both SDDI and IDDI significantly compared to the control. The best results were obtained by the mixture of cumin oil and nigella oil (Fig. 1).

### Biochemical analysis

#### Total anthocyanin content (g/100 g of fresh ray flowers)

The utilized EOs mixtures positively affected the ray flowers’ anthocyanin content compared to the control cut inflorescences. The highest value was recorded with the mixture of lavender oil and thyme oil (Table 2).

**Table 2:** Total anthocyanin content, total carbohydrate content and total plate count of microorganisms in preservative solution affected by different EOs mixtures which were added to the vase solution of *Gerbera Jamesonii* L. c.v. Froza.

Treatments	Total anthocyanin content (g/100 g of fresh petals)	Total carbohydrate content (% of D.W. of the inflorescence)	Total plate count of microorganisms in preservation solution (CFU/ml) (the count × 10^− 6^)
T1	0.048^c^ ± 0.002	17.96^c^ ± 0.55	20.067^a^ ± 0.5207
T2	0.072^a^ ± 0.002	32.91^a^ ± 2.58	0.157^b^ ± 0.0090
T3	0.060^b^ ± 0.001	21.82b^c^ ± 0.64	0.310^b^± 0.0058
T4	0.075^a^ ± 0.001	18.55^c^ ± 0.83	0.440^b^ ± 0.0058
T5	0.076^a^ ± 0.001	24.81^b^ ± 0.67	0.014^b^ ± 0.0006

The means inside a column with the same letters are not significantly different according to Duncan’s Multiple Range Test (at the 0.05% level). [T1]: Control (distilled water and 0.5 µlL^− 1^ of tween-20); [T2]: Cumin oil (150 µlL^− 1^) and Peppermint oil (150 µlL^− 1^); [T3]: Cumin oil (150 µlL^− 1^) and Nigella oil (150 µlL^− 1^); [T4]: Clove oil (150 µlL^− 1^) and Anise oil (150 µlL^− 1^); [T5]: Lavender oil (150 µlL^− 1^) and Thyme oil (150 µlL^− 1^).

#### Total carbohydrate content (percentage Of D.W. Of the inflorescence)

Data presented in Table 2 show statistic differences between treatments. The lowest value obtained by the control while the most positive treatment was the mixture of cumin oil and peppermint oil.

### Biological studies

#### Total plate count of microorganisms in preservation solution [Colony forming unit (CFU/ml)

Data recorded in Table 2 display obviously the effectiveness of EOs mixtures in decreasing significantly the total plate count of microorganisms in the vase solution of gerbera cut inflorescences compared to the control. In addition, the mixture of lavender oil and thyme oil efficiently inhibited the growth of the microorganisms in the vase solution compared to the other treatments, the control as well.

## Discussion

Recently, essential oils effectively expanded the vase life of various cut flowers. Besides, they maintained all aspects of their quality and enhanced the efficiency of their water relations. Furthermore, they effectively reduced the decomposition of pigments and preserve the carbohydrate reserves of the cut flowers. In addition, they influentially inhibited the growth of microorganisms in the preservative solutions of the cut flowers.

All applied EOs mixtures efficiently elongated the vase life of gerbera cut inflorescences, with priority of the mixture of Cumin oil (150 µlL^− 1^) and Nigella oil (150 µlL^− 1^). In the same way, 150 mgL^− 1^ cumin oil with 600 mgL^− 1^ 8-hydroxyquinoline sulfate elongated the vase life of cut rose flowers^16^, while 300 mgL^− 1^ cumin oil recorded the highest increment of vase life of rose cut flowers by Mirjalili, 2023^4^. They attributed the effectiveness of cumin oil to the main component, cumin aldehyde, which acts as a stimulant, as well as its key role as an antimicrobial compound. On the other hand, nigella oil had the best effect on the vase life of solidago cut flowers^6^. The prime constituent of nigella oil is thymoquinone, which is characterized as a strong antimicrobial compound. The antimicrobial effects of EOs are the fundamental reason for prolonging the vase life of the cut flowers, due to decreasing the occlusion of the vessels caused by the microorganisms.

The mentioned mixture (Cumin oil and Nigella oil) also statistically increased the R.F.W. % and the D.M. %, rather than the control and other treatments. Cumin oil efficiently maintained the fresh and dry weight of lily cut spikes either alone or in combination with peppermint oil^17,18^. Likewise, nigella oil at 200 mgL^− 1^ recorded the highest values of the R.F.W. % and the ratio between fresh weight and dry weight of solidago cut flowers^6^.

Although the mixture of cumin oil and peppermint oil resulted in the highest water uptake and water loss, the mixture of cumin oil and nigella oil recorded values of total and daily water uptake (71.25 ml inflorescence^− 1^ and 4.06 ml day^− 1^ inflorescence^− 1^) higher than its values of total and daily water loss (67.58 g inflorescence^− 1^ and 3.85 g day^− 1^ inflorescence^− 1^), and thus created a positive balance between the absorbed water and the lost water. Cumin oil with 8-hydroxyquinoline sulfate positively enhanced the relative water content of rose cut flowers^16^. Furthermore, cumin oil with peppermint oil improved the water relations of lily cut inflorescences^17^. The essential oils efficiently reduced metabolism associated with a reduction in transpiration, improving water relations and hydraulic conductance, maintaining carbohydrates, resulting in an increase in water uptake, and preserving the fresh and dry weights of cut flowers.

All applied mixtures significantly increased the diameter decreasing index of the inflorescence and the scape. Cumin oil (150 µlL^– 1^) and Nigella oil (150 µlL^– 1^) had the best results. Cumin oil at 150 mgL^− 1^ with 400 mgL^–1^ 8-hydroxyquinoline sulfate increased the flower diameter of rose cut flowers^16^. Cumin oil at 150 µlL^– 1^ with peppermint oil at 150 µlL^– 1^ recorded the highest values of stalk diameter of lily cut inflorescences^18^. The antimicrobial effects of essential oils protect vessels from microbial blockage, so they raised the amounts of solution uptake and thus the flower and the stem diameters increased^19^.

The utilized EOs effectively preserved the total contents of anthocyanins and the carbohydrates in comparison with the control. These results agree with those of former researchers. Cumin oil increased both the total anthocyanin content and the soluble sugar content of rose cut flowers^4^. Thyme oil efficiently maintained the chlorophyll and the carbohydrate contents of carnation cut flowers^8^. On the other hand, peppermint oil enhanced the anthocyanin content of *Dendrobium* orchid cut flowers^5^. The essential oils are capable of suppressing the growth of microorganisms. Growth of microorganisms in the vase solution is responsible for producing the ethylene hormone. Therefore, the EOs indirectly decrease the amount of ethylene and delay the degradation of the flowers’ pigments. In addition, essential oils possess antioxidant properties through the reduction of water stress and obstruction of vessels. The increase of oxygen-free radicals in chloroplasts destroys pigment molecules and membranes of chloroplasts. The use of EOs with their anti-radical properties maintains the pigments in cut flowers^18^.

The mixtures of essential oils effectively reduced the count of microorganisms in the vase solution rather than the control. Lavender oil with thyme oil prevents microbial development in the vase solution of gerbera cut inflorescences. Thyme oil was so effective in reducing the evolution of microbes in the vase solution of lily cut inflorescences; however, thyme oil in combination with cinnamon oil was more efficient in suppressing the growth of the microorganisms^18^. The antimicrobial mechanism of EOs was attributed to the inhibition of the synthesis of DNA, RNA, protein, and polysaccharides. These antibacterial properties may also be due to the high levels of phenolic compounds such as carvacrol, thymol, and eugenol. It was also reported that EOs lowered the pH of the vase solutions, therefore limiting the growth of bacteria^5^.

## Conclusion

In the last two decades, essential oils have efficiently prolonged the shelf life of many cut flowers while preserving their quality, inner constituents, and the purity of their vase solution. Therefore, this investigation was conducted to benefit from the affirmative effects of EOs by applying some mixtures of them. The tested mixtures were: (a) cumin oil and peppermint oil, (b) cumin oil and nigella oil, (c) clove oil and anise oil, and (d) lavender oil and thyme oil, at the concentration of 150 µlL^− 1^ for each one. Their impacts were examined on *Gerbera jamesonii* L. cv. Froza cut inflorescences. The results indicated the efficacy of the utilized mixtures in elongating the shelf life of gerbera cut inflorescences. In addition, they maintained their fresh and dry weights, enhanced the water relations, raised both the diameters of the inflorescence and scape, and preserved the anthocyanin and carbohydrate contents. They also dramatically decreased microbial evolution. In most traits, the combination of cumin oil and nigella oil was the most effective mixture. This investigation requires further studies and analysis to examine as many possible mixtures at different concentrations.

## Data Availability

The datasets generated and/or analyzed during the current study are included in this published.
